# Matters Arising ‘Lewy body disease or diseases with Lewy bodies?’

**DOI:** 10.1038/s41531-022-00337-4

**Published:** 2022-06-23

**Authors:** Kurt A. Jellinger

**Affiliations:** Institute of Clinical Neurobiology, Vienna, Austria

**Keywords:** Parkinson's disease, Neurodegeneration

**arising from** Kateřina Menšíková et al. *npj Parkinson's Disease* 10.1038/s41531-021-00273-9 (2022)

Menšíková et al.^1^ in their recent review of Lewy body disease, emphasized (a) that from the strict pathological point of view, there is practically no difference between PD and PDD, even the experienced neuropathologist is not able to differentiate; and (b) that there is no sharp pathological border between PDD and DLB, although the degree of AD pathology and the presence of CAA are probably the most significant pathological differences between these two phenotypes. These two statements need some discussion based on personal and other recent data about the complex relations between cognitive impairment and neuropathology in LB diseases. Ad (a): In a personal autopsy series of 330 PD patients, only 3.2% of demented ones (37.6% of the total cohort) had Braak LB stages 2–3 with severe AD co-pathology (neuritic Braak stages V and VI), while 35.5% of demented PD patients revealed LB stages 4 or 5 with superimposed neuritic Braak stages V and VI. More than 50% of them showed a strong relationship between the severity of αSyn and tau pathologies, particularly in the limbic system. LB pathology with moderate or high grade AD lesions was seen in 40% of PDD patients, while one third of cases with diffuse LB pathology and mild AD lesions, restricted to Aβ plaques of limbic tau pathology, did not show considerable cognitive impairment^2^. PDD patients had significantly lower brain weight than non-demented ones, while significantly more severe Alzheimer neuropathological changes were present in PDD than non-demented (ND) PD patients^3^. In another study of 60 ND-PD and 110 PDD patients, the latter were significantly older than ND-PD ones—83.9 vs. 77.8 years; *p* < 0.01), PDD showed only slightly higher Braak LB scores (mean 4.2 vs. 4.0), but significantly higher neuritic Braak stages (mean 5.2 vs. 4.4), Thal Aβ phases (mean 3.0 vs. 2.3), and both significantly higher CAA frequency and severity (50% vs. 21.7% and mean 0.72 vs. 0.26). In conclusion, association between cortical Aβ load, generalized CAA and tau pathology are the morphological basis of cognitive decline in PD^4^.

Ad (b): There is general agreement that both PDD and DLB share neuropathological features, with a variable mixture of LB and AD-related co-pathologies. However, recent studies have shown some essential morphological differences between PDD and DLB, that is, more frequently increased cortical and striatal Aβ load^5^, more severe cortical tau pathology (Braak 5.1 ± 0.7 vs. 4.2 ± 0.4), and higher CAA frequency and severity (91% vs. 50% and 2.3 ± 0.2 vs. 0.72 ± 0.2; both *p* < 0.01), the latter mainly in occipital and less in frontal lobes^4,6^. Most frequent and highest CAA scores were seen in cases with APOE ε4 (PDD 33.3%, DLB 48%)^7^. Cortical tau pathology was also more frequent in DLB, the incidence of negative cases being 70% vs. 82% in PDD, also supporting the notion that the morphological distinction between the two phenotypes is not restricted to Aβ deposition, cortical LB and tau pathologies^8^. Further differences are more severe αSyn load in hippocampal subareas CA 2/3 and entorhinal cortex (EC) implicating the role of the EC-CA circuitry in the pathogenesis of DLB^9^. There is different involvement of substantia nigra, with more severe neuronal loss in ventrolateral cell groups in PD, but predominant damage of dorsolateral ones in DLB^10^, causing less severe postsynaptic dopaminergic upregulation, while significantly higher 5-HT1A receptor-binding density in cortex is seen in DLB compared to PDD^11^. DLB showed worse prognosis than PDD (mean survival mean 6.7 vs. 12.5 years; *p* < 0.01), which was linked to both increased tau and CAA pathologies distinguishing both disorders. The reasons for these clinical and pathological differences between the phenotypes, PD, PDD and DLB(+AD) are not clear, but recent studies suggest a close interaction of the various pathological proteins, in particular between αSyn, Aβ and tau^12^ within the spectrum of LB diseases (or diseases with LBs)^13^. Cerebrovascular co-pathologies, in particular cerebral microbleeds, showed a similar prevalence in PDD, DLB and AD^14^, although recent studies suggested a synergistic interaction between cerebrovascular disease and Lewy pathologies, which possibly extends to other highly concomitant pathologies in LB disorders^15^. In conclusion, it should be emphasized that other co-pathologies may influence the clinical features and progression in both PDD and DLB^16^. The impact of all of them, however, needs further elucidation.
